# Primary rhabdoid epithelioid sarcoma of the left thigh mimicking epithelioid rhabdomyosarcoma: A diagnostic pitfall

**DOI:** 10.1016/j.ijscr.2020.04.002

**Published:** 2020-05-08

**Authors:** Rahadyan Magetsari, Ery Kus Dwianingsih, Yuni Artha Prabowo Putro, Irene Araneta, Yudha Mathan Sakti

**Affiliations:** a Department of Orthopaedics and Traumatology, Faculty of Medicine Universitas Gadjah Mada – Dr. Sardjito General Hospital, Yogyakarta, Indonesia; b Department of Anatomic Pathology, Faculty of Medicine Universitas Gadjah Mada – Dr. Sardjito General Hospital, Yogyakarta, Indonesia

**Keywords:** Epithelioid sarcoma, Bone tumor, Examination

## Abstract

**Introduction:**

Epithelioid sarcoma (ES) is a rare mesenchymal tumor, accounting for less than 1% of all adult soft-tissue sarcomas. The diagnosis of such malignancy is challenging. We reported a 31-year-old male diagnosed with rhabdoid ES that histologically mimicked epithelioid rhabdomyosarcoma in a 31-year-old male.

**Case presentation:**

A 39-year-old male presented with pain in the left thigh. He had planned for open reduction internal fixation due to left femur fracture. During the surgery, the surgeon found abnormal appearance of the bone and the surrounding tissue. The histopathology examination showed spindle-to-polygonal epithelioid cells arranged in nodular pattern with necrotic areas. Immunohistochemistry examination revealed vimentin and CK positive expression, while myogenin was negative. Further immunostaining using CD34 and HMB45 were also negative.

**Discussion:**

The diagnosis of ES based on only the clinical manifestation has proven to be difficult. Thus, the histopathology examination followed by immunohistochemistry is considered as the main modality for the diagnosis. Better understanding of clinical properties of ES will aid in deciding the best treatment for the patient.

## Highlights

• The diagnosis of epithelioid sarcoma based on only the clinical manifestation has proven to be difficult.
• Thus, the histopathology examination followed by immunohistochemistry is considered as the main modality for the diagnosis.
• Better understanding of clinical properties of epithelioid sarcoma will aid in deciding the best treatment for the patient.

## 1 Introduction

Epithelioid sarcoma (ES) is a rare mesenchymal tumor that occurs mostly in the dermal or subcutaneous area of the distal extremities. Generally, ES is considered as a high-grade soft tissue sarcoma [1]. ES occurs in less than 1% of all adult soft-tissue sarcomas and approximately 4%–8% of pediatric non-rhabdomyosarcomatous sarcoma [2,3]. Due to the rare prevalence of ES, it is hard to understand its clinical manifestation. This tumor frequently recurs at the same location and can undergo metastasis to lymph nodes, soft tissues, lungs, brain, or bones. ES tends to be multifocal at the first occurrence and during the recurrence [1,4]. Histologically, there is a diagnostic pitfall in diagnosing between a rhabdoid epithelioid sarcoma and an epithelioid rhabdomyosarcoma. This work has been reported in line with the SCARE criteria [5].

## 2 Case report

**Fig. 1:**
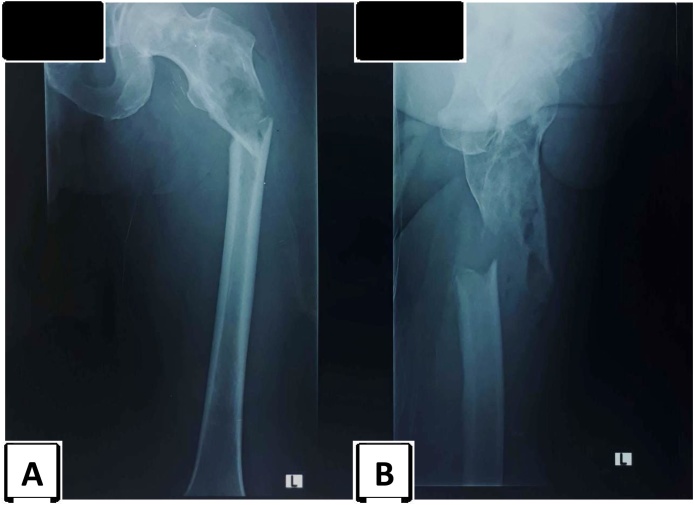
Radiographic pictures. Anteroposterior (A) and lateral (B) projections showed lytic lesion in the area of subtrochanter of the left femur with pathological fracture and soft tissue swelling.

A 39-year old male presented with pain and discomfort of the left thigh. One month prior to the admission, the patient complained of discomfortness with intermittent pain in his left thigh and went to a general practitioner. However, the pain did not subside. Six days before the admission, the patient felt sudden pain in the left thigh while walking fast, then he fell down and was unable to walk. He was brought to a private hospital in Yogyakarta and X-ray examination was performed, showing a fracture and soft tissue mass in the left proximal femur (Fig. 1). The surgeon decided to perform open reduction and internal fixation (ORIF). During the surgery, abnormal appearance of the bone and the surrounding tissue was found. Tissue samples were taken for histopathology examination and skeletal traction was performed instead of ORIF. The patient was then referred to our institute for further investigation.

Laboratory examination showed normal range of complete blood count, electrolytes, alkaline phosphatase (ALP), and also lactate dehydrogenase (LDH). There was no increased levels in other specific tumor marker examinations of other organs which showed no source of metastatic bone disease. Bone survey was performed and showed no sign of abnormality in other bones. Further, computerized tomography (CT) scan of the chest and abdomen were done and also showed no signs of metastasis in the regions. Magnetic resonance imaging (MRI) was done. It showed the presence of soft tissue lesion in the left proximal femur (subtrochanteric) with the size 12.2 × 9.8 × 13.8 cm, with expansion of the lesion to femoral head, femoral neck, trochanteric area and femoral shaft causing pathologic fracture of the left subtrochanter femur.

**Fig. 2:**
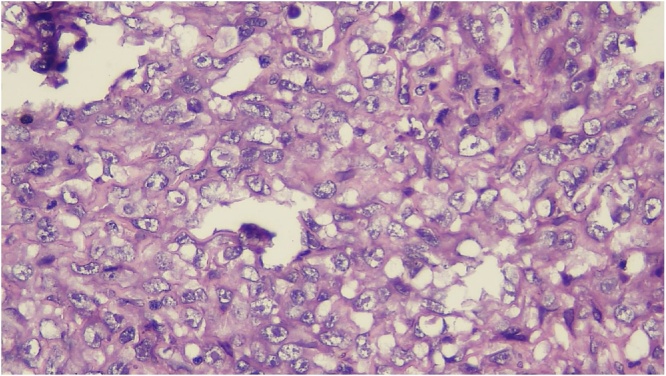
Mesenchymal tumor epitheloid shape with increased cellularity, arranged in solid and alveolar fashion and infiltrative to surrounding soft tissue. (HE, ×40).

**Fig. 3:**
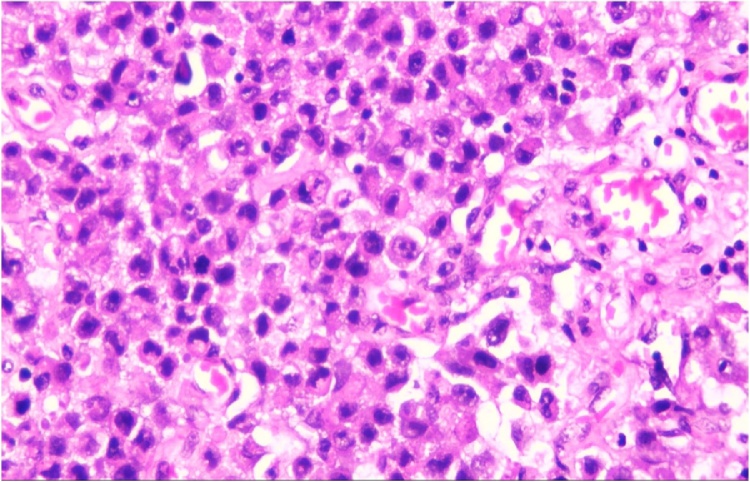
Epitheloid cells, small to moderate in size, eosinophilic cytoplasm, with caudated appearance mimicking rhadomyoblast. Round to oval nuclei, some spindle nuclei are found with irregular chromatin, and prominent nucleoli. (HE, ×400).

**Fig. 4:**
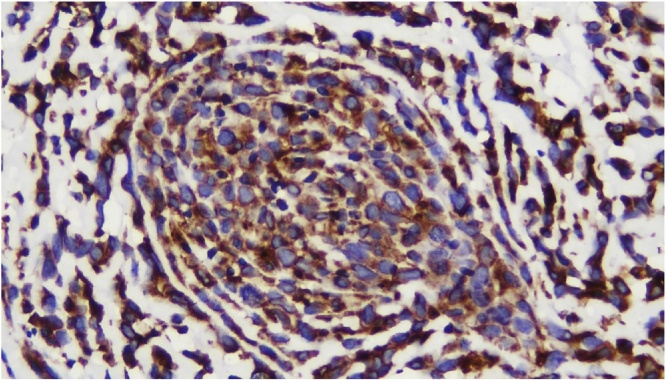
Strong diffuse positive stained with vimentin, ×400.

**Fig. 5:**
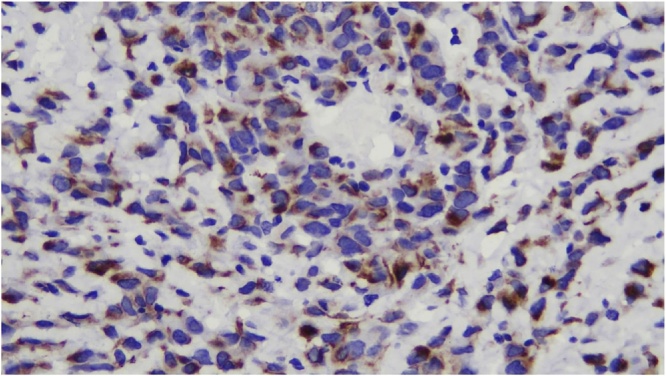
Strong diffuse positive stained with CK, ×400.

**Fig. 6:**
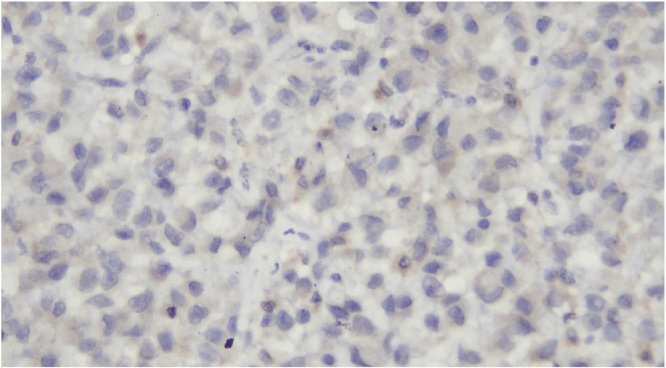
Negative stained with myogenin, ×400.

**Fig. 7:**
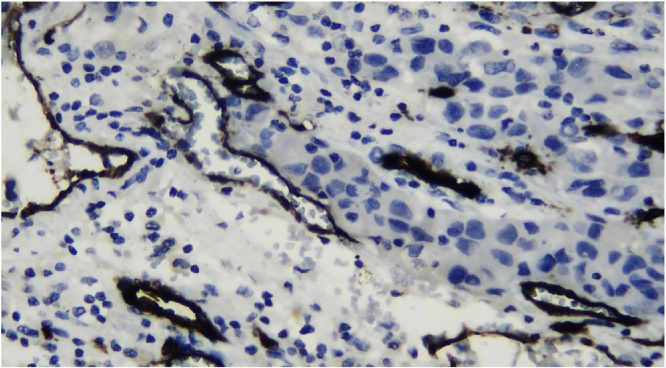
Positively stained with CD34 in blood vessel cells membrane, negatively stained in tumor cells, ×400.

**Fig. 8:**
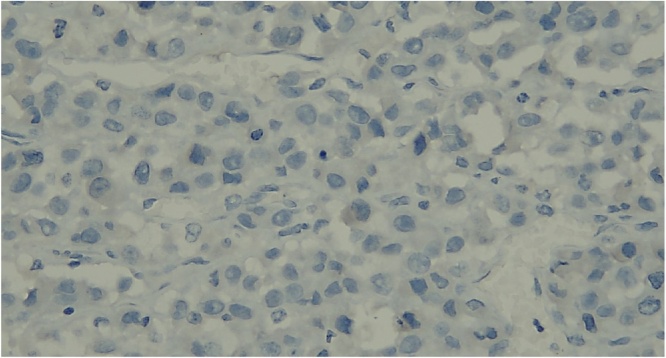
Negatively stained with HMB45, ×400.

Histologic section showed mesenchymal tumors with increased cellularity, arranged in solid and alveolar fashion and infiltration to surrounding soft tissue (Fig. 2). Moreover, the tumor cells consisted of epithelioid cells, small to moderate in size. Eosinophilic cytoplasm was visible with caudated appearance and round to oval nuclei, while some spindle nuclei were found with irregular chromatin, and prominent nucleoli (Fig. 3). Numerous mitosis and a wide area of necrosis were present. Based on morphology, the tumor was assessed as epithelioid rhabdomyosarcoma with differential diagnosis of epithelioid sarcoma. Immunohistochemistry examination revealed vimentin (Fig. 4) and CK positive expression (Fig. 5), while myogenin was negative (Fig. 6). Further immunostaining using CD34 (Fig. 7) and HMB45 were also negative (Fig. 8). Thus, immunostaining profile was suitable as proximal epithelioid sarcoma, with the rhabdoid variant.

A radical resection was planned, and the patient was taken for surgical hip disarticulation. Following the surgery, adjuvant chemotherapy was done using Doxorubicin and Ifosfamide for 6 cycles. Then, the patient was referred to radiation oncology and underwent intensity-modulated radiation therapy at 6000 cGy to the left hip joint.

The patient had a slow yet uneventful postoperative recovery. On the most recent CT imaging of chest and abdomen done 1-year postoperatively, there was no evidence of metastasis. He returned to daily activities using a lower limb prosthesis and remained free of recurrence.

## 3 Discussion

Epithelioid sarcoma (ES) is a rare soft tissue sarcoma mostly affecting the age group of 20–40 years old. Overall peak incidence of ES is 35 years of age. It was first clearly characterized by F.M. Enzinger in 1970. The incidence is only less than 1% of all adult soft tissue sarcoma and between 4–8% of pediatric non-rhabodmyosarcomatous sarcoma [6]. ES is a rare, slow-growing sarcoma with a high risk of recurrence and metastasis. Due to the slow growth of the tumor, rarity and lack of symptoms, the diagnosis of ES is very challenging. Because of this, it is almost impossible to perform a large number of clinical trials to evaluate different treatment modalities.

ES is divided into two clinicopathological entities, which are distal-type ES and proximal-type ES. These two subtypes are considered as a continuum of this disease, not as distinct entities. Distal-type ES tends to be found in younger age predominantly male patients, compared to proximal-type [7]. ES also had been reported to occur in the iliac bone [8]. About 47% of ES patients exhibit localized disease. However, some patients present with multiple nodules and metastatic lesions.

On physical examination, distal-type ES is characterized as a firm, non-tender, slow-growing tumor with predilection towards distal extremities, especially the hands [9]. Different from distal-type, proximal-type ES often is diagnosed as deep, infiltrating soft tissue masses. Further, proximal type ES is also associated with a more aggressive course than distal-type.

Distal-type ES or classic ES is characterized as tumor nodules with central necrosis and surrounded by large polygonal cells and spindle cells in the periphery. This type of ES includes angiomatoid, fibroma-like, and myxoid variants. Whereas, proximal-type ES is characterized by a tumor with multinodular pattern and sheet-like growth of large polygonal cells. This type is often accompanied by a focal or predominant rhabdoid morphology [6].

In this case, the tumor was in the proximal femur as a soft tissue lesion and infiltrating surrounding deep soft tissue. The tumor consisted of spindle to polygonal epithelioid cells arranged in nodules, small to moderate in size, with a wide necrotic area. Also, there was abundant pathological mitosis, which showed an aggressive course. The tumor was more suitable to be considered as proximal type ES based on the physical examination and tumor cell morphology.

Due to its wide range of morphology, it is hard to make definitive diagnosis of ES based on histopathology examination alone. Given the epithelioid morphology of the tumor cells, the differential diagnosis of this case was epithelioid rhabdomyosarcoma which can show almost identical histological patterns. To aggravate this challenge, there is no specific ES biomarker that has been identified. Both of these tumors also express epithelial markers such as cytokeratin in immunohistochemistry. But co-expression of vimentin and keratin is thought to be characteristic of ES. CD34 is positively expressed in 50–60% cases of ES while, it is negatively expressed in rhabdomyosarcoma.9 Also, myogenin can help to distinguish ES from rhabdomyosarcoma and HMB45 can help to distinguish ES from melanoma cases [8].

Regarding this case, the immunohistochemistry examination showed that all tumor cells were positively stained with vimentin and CK but negatively stained with myogenin, which excluded the diagnosis of rhabdomyosarcoma. However, further immunostainings using CD34 and HMB45 were also negative, which showed that the tumor was suitable as epithelioid sarcoma of the bone.

Currently, there is no optimal management of ES, and it rarely spreads to lymph nodes. Local recurrence can occur within 1–2 years after treatment. Moreover, most of these cases were often accompanied by distant metastasis [10]. It is reported that local relapse rate is about 35% [11]. It has also been reported that ES can metastasize widely to lymph nodes, skin, scalp, brain, digestive tract, liver, kidneys, and musculoskeletal system [10].

The primary treatment is wide surgical resection for localized disease, and the goal of the treatment is to remove the tumor while maintaining the optimal function of the extremity [9,10]. In this case, hip disarticulation was performed because the cancer was in the proximal femur with expansion of the lesion to the femoral head. The treatment goal was to obtain a tumor-free margin to reduce the possibilities of local recurrence and distant metastasis.

Adjuvant radiation therapy after resection can be employed to reduce local recurrence [12,13]. Radiation can also be considered for patients with marginal primary resection, local-regional recurrence, or palliative treatment [9]. The use of chemotherapy is particularly indicated in metastasis cases [10].

Patients with localized disease have a better 5-year overall survival at about 75%. Metastasis occurs in almost half of the patients with the localized disease even with adequate treatments. The width of the tumor has been reported to be directly proportional to the rate of metastasis and lower 10-year survival. Female gender is linked with a better prognosis, while tumor size, vascular invasion, and necrosis have been inconsistently reported with a poorer prognosi [10]. Proximal lesions indicate worse prognosis as compared to distal lesions. Patients with tumor located proximal to the elbow or knee have poorer prognosis due to its unfavorable features, such as increased width, and involvement of the pelvic, perineal, and genital region [3]. Patients with metastasis have a poor prognosis; reported median survival rate is 32–52 weeks [2,9]. The 1-year survival rate and 5-year survival rate are 46% and 0% [2,14].

Wide surgical excision remains the mainstay of treatment, and medical management for systemic disease is, to a large extent, undefined. For localized disease, neoadjuvant or adjuvant radiation therapy is often given to reduce local relapse, but the role of adjuvant chemotherapy is unclear. Systemic chemotherapy provides satisfactory palliation, but response is of short duration. As relapse rates remain high despite multimodal therapy, there is an urgent need for development of targeted treatments for patients with regional and distant metastases [15].

## 4 Conclusion

There are no distinct clinical properties of epithelioid tumors to differentiate with other tumors. Clinically, it is essential to distinguish between the primary bone or soft tissue tumor and metastatic tumor. Radiology examination will help to distinguish metastatic carcinoma. Further, histopathology examination followed by immunohistochemistry will help to establish the diagnosis. Better understanding of the clinical properties of ES will facilitate the surgeon with a better choice of treatment. Complete resection while maintaining optimal function of the extremity is considered as the main treatment. Poorly-planned biopsy of a primary malignancy may contaminate the field, leading to loss of options in considering limb salvage surgery.

## Declaration of Competing Interest

None.

## Sources of funding

None.

## Ethical approval

Ethical approval was received from the Ethical Committee of Faculty of Medicine, Universitas Gadjah Mada.

## Consent

Written informed consent was obtained from the patient for publication of this case report and accompanying images. A copy of the written consent is available for review by the Editor-in-Chief of this journal on request.

## Author contribution

Rahadyan Magetsari: performing the procedure, study concept, data collection.

Ery Kus Dwianingsih: data collection, data interpretation, writing the paper.

Yudha Mathan Sakti: writing the paper.

Yuni Artha Prabowo Putro: writing the paper.

Irene Araneta: writing the paper.

## Registration of research studies

This study has been registered at researchregistry.com (UIN: researchregistry5162).

## Guarantor

Rahadyan Magetsari.

## Provenance and peer review

Not commissioned, externally peer-reviewed.
